# Characterization of Antibiotic Administration Factors Associated with Microbiome Disruption and Subsequent Antibiotic-Resistant Infection and Colonization Events in Acute Myeloid Leukemia Patients Receiving Chemotherapy

**DOI:** 10.3390/antibiotics14080770

**Published:** 2025-07-30

**Authors:** Samantha Franklin, Corina Ramont, Maliha Batool, Stephanie McMahon, Pranoti Sahasrabhojane, John C. Blazier, Dimitrios P. Kontoyiannis, Yang Ni, Jessica Galloway-Peña

**Affiliations:** 1 Interdisciplinary Graduate Program in Genetics and Genomics, Texas A&M University, College Station, TX 77843, USA; srfranklin@tamu.edu (S.F.); stephmcm@tamu.edu (S.M.); 2 Department of Statistics, Texas A&M University, College Station, TX 77843, USAyni@stat.tamu.edu (Y.N.); 3 Department of Veterinary Pathobiology, College of Veterinary Medicine and Biomedical Sciences, Texas A&M University, College Station, TX 77843, USA; 4 Department of Infectious Disease, Infection Control and Employee Health, The University of Texas MD, Anderson Cancer Center, Houston, TX 77030, USA; pvsahasrabhojane@mdanderson.org (P.S.); dkontoyi@mdanderson.org (D.P.K.); 5 Texas A&M Institute for Genome Sciences & Society, Texas A&M University, College Station, TX 77843, USA; jcb244@tamu.edu

**Keywords:** microbiome, resistome, antibiotic resistance, colonization, infection, leukemia

## Abstract

Background: Broad-spectrum antibiotics are often used for suspected infections in patients with hematologic malignancies due to the risk of severe infections. Although antibiotic use can lead to antimicrobial resistance and microbiome dysbiosis, the effects of antibiotics on the microbiome and resistome in patients with acute myeloid leukemia (AML) undergoing remission induction chemotherapy (RIC) are not well understood. Methods: Various statistical models were utilized to examine the effects of antibiotic administration on the microbiome and resistome over time, as well as differences in AR-infection (ARI) and colonization (ARC) by important CDC-threats in 119 AML patients. Results: A greater number of unique antibiotic classes administered correlated with a loss of unique antibiotic resistance genes (ARGs) (R = −0.39, *p* = 0.008). Specifically, although a greater number of oxazolidinone administrations was correlated with a greater loss of diversity (R = −0.58, *p* < 0.001), each additional day of linezolid reduced the risk of ARC by ~30% (HR: 0.663, *p* = 0.047) and decreased the odds of acquiring genes predicted to confer macrolide (HR: 0.50, *p* = 0.026) resistance. Conclusions: The number of antibiotic administrations and the types of antibiotics used can influence the risk of antibiotic resistance gene (ARG) expansion and ARC events in AML patients undergoing RIC. While certain antibiotics may reduce microbial diversity, they are not always linked to an increase in ARGs or ARC events.

## 1. Introduction

The prevalence of antibiotic-resistant bacteria poses a serious threat to the efficacy of commonly used antibiotics against a growing number of bacterial infections, requiring immediate intervention [1,2]. Furthermore, the use of broad-spectrum antibiotics provides selective pressure on microbial communities, thus favoring the emergence of resistant bacterial strains [3]. The burden of antibiotic resistance is of particular significance for patients with hematological malignancy, who are at a very high risk of acquiring severe, often life-threatening infections [4]. The routine use of fluoroquinolone prophylaxis before and during chemotherapy, as well as broad-spectrum empirical therapy upon neutropenic fever, has been established as a standard of care for heme malignancy patients receiving cytotoxic chemotherapy [5,6]. However, the prolonged use of antibiotics may disrupt the microbial communities and facilitate the accumulation of antimicrobial resistance genes (ARGs) in the gut microbiome of these patients [7,8,9]. Furthermore, treatment with chemotherapeutic drugs may damage the gut mucosa by increasing the intestinal permeability [10,11]. Consequently, both scenarios increase the risk of bacterial translocation of potentially antibiotic-resistant (AR) bacterial species across the gut wall, causing difficult-to-treat systemic infections in these patients [10,11]. Although colonization and infection with AR pathogens in cancer patients are common, there is a paucity of microbiome data regarding the associated risk factors of AR infectious toxicities during remission induction chemotherapy (RIC).

In 2019, the Centers for Disease Control and Prevention (CDC) published a report on antibiotic resistance threats. In this report, specific bacteria are classified into urgent, serious, and concerning AR-threats in the United States [12]. Among them are organisms such as multidrug-resistant *Pseudomonas aeruginosa* (MDRP), extended-spectrum beta-lactamase-producing Enterobacteriaceae (ESBL), methicillin-resistant *Staphylococcus aureus* (MRSA), vancomycin-resistant *Enterococcus* (VRE), and carbapenem-resistant Enterobacteriaceae (CRE). Acute leukemia patients are at particularly high risk of developing infections with these same organisms [13]. Specifically, among multi-center studies, it has been shown that *Escherichia coli*, *Klebsiella pneumoniae*, *Pseudomonas aeruginosa*, coagulase-negative staphylococci, *Staphylococcus aureus*, and enterococci account for the vast majority of bacteremia episodes in febrile neutropenia patients [14,15,16]. Extended-spectrum β-lactamase (ESBL)-producing Enterobacteriaceae have accounted for 20–50% of Enterobacteriaceae-related bloodstream infections in neutropenic patients, while colonization with CRE can be up to 45% in neutropenic patients [16]. Although Gram-positive infections have decreased over time, while multidrug-resistant Gram-negative infections are on the rise in heme malignancy patients, MRSA and VRE are the predominant Gram-positive species causing infections, in addition to viridans group streptococci and coagulase-negative staphylococci [17].

While AR pathogens are of great concern, the gut resistome is of particular significance as it is considered a key reservoir of ARGs that can no longer be overlooked when assessing AR complications in cancer patients [18,19]. Given that the commensal bacteria residing in the gut contain a large reservoir of ARGs (collectively termed as the resistome) [20], ARGs can be transferred among bacterial species within the microbiome via horizontal gene transfer, presenting serious consequences for hospitalized patients [21,22,23]. Bacteria may carry ARGs that confer resistance to a single antibiotic or mobile genetic elements (MGE) that confer resistance to several antibiotics [24]. Thus, increasing the selection for and number of bacteria harboring ARGs or MGEs containing ARGs within the microbiome amplifies the resistome, resulting in difficult-to-treat infections [25].

Given that ~60–80% of acute myeloid leukemia (AML) patients will experience febrile neutropenia [5,26], many are treated with broad-spectrum antibiotics for suspected infection, which may change the bacterial diversity and the frequency of ARGs in the gut [27]. While febrile neutropenia should be treated urgently as a potential infection, this condition may not always indicate a microbial infectious cause. Our research group has previously demonstrated that AML patients who experienced loss of microbial diversity over the course of RIC were significantly more likely to contract a microbiologically documented infection. The same published study suggested that loss of stool bacterial diversity in AML patients was significantly correlated with the receipt of carbapenems [27]. In a separate study, we observed that cumulative antibiotic exposure was significantly associated with microbiome (α-diversity) temporal variability and subsequent infection [28]. While it is well known that the use of antibiotics contributes to the emergence of drug resistance and microbiome disruption, there is minimal understanding of how antimicrobial use in treated hematologic malignancy patients impacts both the microbiome and resistome. We hypothesize that the duration and type of antibiotic exposure are important for the risk of ARG expansion, AR-threat colonization (ARC), and AR infection (ARI) in AML patients undergoing induction chemotherapy. Thus, in this study, we analyzed prior antibiotic exposures and their association with colonization or infection events with CDC AR-threat pathogens in AML patients receiving RIC. We also employed shotgun metagenomics data from 119 AML patients to investigate the effect of antibiotic administration on the abundance and gene ontology of ARGs in the gut microbiome over time. Specifically, we included the variables of administration numbers and antibiotic type to determine whether specific administration variables were associated with AR-threat outcomes, the loss of microbial diversity, and ARG expansion.

## 2. Results

### 2.1. The Impact of Total Antimicrobial Administrations on Patient AR-Threat Outcomes, Resistome, and Microbial Diversity

A total of 168 stool samples were included in the metagenomic analyses, including 98 BL samples and 70 EOS samples, representing 119 patients with complete metagenomic, clinical, and pharmacologic data available (Table 1). Eight patients had a confirmed infection with an AR-threat pathogen (ARI), with MDRP being the most predominant ARI pathogen (50% of the isolates). Fourteen patients were confirmed to have gastrointestinal colonization with an AR-threat pathogen (ARC), with VRE accounting for 1/3 of those isolates.

The antibiotic class administered to the most patients was fluoroquinolones (~85%), as expected, as this is the standard prophylaxis. This was followed by oxazolidinones in ~75% of patients, and cephalosporins in ~74% of patients. This is also to be expected as cefpodoxime is used for prophylaxis if a fluoroquinolone (namely levofloxacin) cannot be used for reasons such as intolerance or drug–drug interactions. Moreover, cefepime is typically the first-line empirical therapy of choice in the event of unspecified neutropenic fever. Linezolid is generally given in combination with cefepime to cover resistant Gram-positive organisms (e.g., MRSA, VRE, viridians group streptococci, etc.) while cultures are pending.

**Figure 1:**
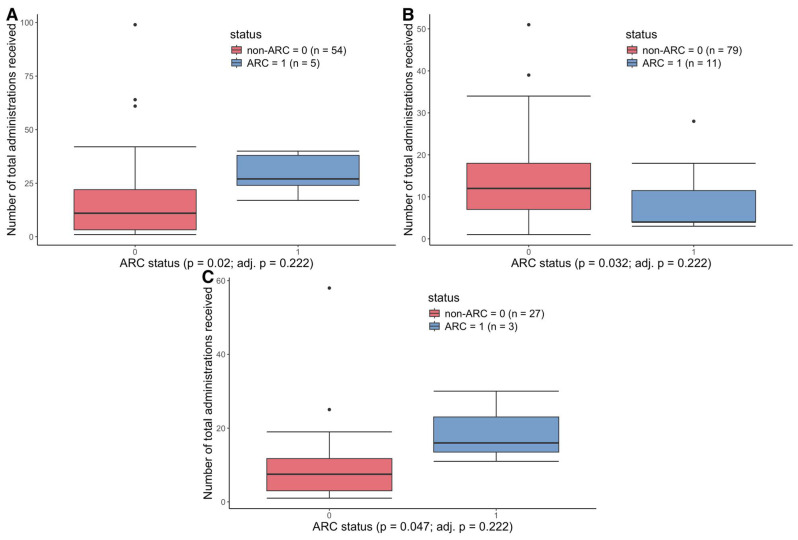
The total number of antibiotic administrations among AR-colonization outcome groups. Of the patients who received (**A**) carbapenem administrations (**B**) oxazolidinone administrations, and (**C**) tetracycline administrations, the total administrations of each antibiotic were compared between patients who experienced colonization with an AR-threat pathogen (ARC) in blue versus patients who did not (non-ARC) in red. Boxplots show the median and interquartile range, with whiskers showing the minimum and maximum, with outliers shown. The number of patients in each group that received each antibiotic is shown in the top right-hand corner of the panel. *p* values are derived from the Mann–Whitney U test with Benjamini–Hochberg adjustment.

We first investigated the effect of the total number of administrations of each class of antibacterial on patient CDC AR-threat outcomes, where each dispensing of a particular drug (regardless of day or dose) counted as one administration (Figure 1, Table S1). When testing if there was a significant difference in the number of total administrations of specific antibiotics among outcome groups (non-ARI vs. ARI, non-ARC vs. ARC, no AR-threat event vs. any AR-threat event), we found higher total administrations of oxazolidinones among patients who did not experience colonization with an AR-threat pathogen, with a median of 12 administrations of oxazolidinones compared to patients who were colonized with an AR-threat pathogen, with a median of 4 administrations (*p* = 0.032, adj = 0.222) (Figure 1B, Table S1). On the other hand, we found higher total administrations of carbapenems (*p* = 0.02, adj = 0.222) and tetracyclines (*p* = 0.047, adj = 0.222) among patients who were colonized with an AR-threat pathogen with a median of 27 and 16 administrations compared to 11 and 8 administrations among patients who did not experience colonization with an AR-threat pathogen, respectively (Figure 1A,C, Table S1).

We also examined the impact of total administrations of each antibiotic class on the ARG differences between baseline (BL) and the end of study (EOS), and identified a higher number of oxazolidinone administrations (median 16 vs. 7 dispenses) among patients who lost unique ARGs between BL and EOS (*p* = 0.03, adj = 0.384) (Table S2) but did not identify correlations with numerical change in unique ARGs between BL and EOS (Table S3). Although these trends were observed, they were not significant when the *p*-value was adjusted for false discovery.

**Figure 2:**
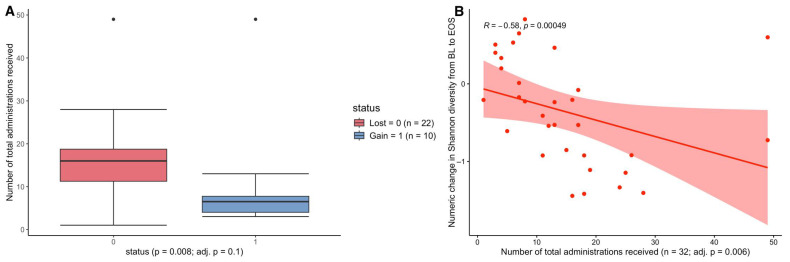
The impact of total administrations and number of days on oxazolidinones on the change in Shannon Diversity from BL to EOS. (**A**) Total oxazolidinone administrations among patients who received oxazolidinone and experienced a loss (red) or gain (blue) in Shannon diversity between BL and EOS. Boxplots show the median total oxazolidinone administrations, interquartile range, and the whiskers are the minimum and maximum values, with outliers shown. *p* values are derived from the Mann–Whitney U test and adjusted utilizing the Benjamini–Hochberg method. (**B**) The numeric change in Shannon diversity from BL to EOS vs. total oxazolidinone administrations among patients who received oxazolidinone. The correlation coefficient (R) and *p* values are derived from Spearman’s correlation rank test and adjusted using the Benjamini–Hochberg method. A total of 32 patients with paired Shannon diversity data at BL and EOS received at least one dose of linezolid while on study.

Given that we had previously observed that antibiotic exposure was significantly associated with α-diversity intra-patient temporal variability, and subsequent infection [28], we also assessed the influence of total administrations of each antibiotic on the differences in Shannon diversity. When comparing the total number of administrations for each antibiotic class amongst patients who gained or lost Shannon diversity between BL and EOS, we found higher total administrations of oxazolidinone (*p* = 0.008, adj = 0.1) in patients who experienced a loss in Shannon diversity with a median of 16 administrations compared to patients who had a gain in Shannon diversity who had a median of 6 administrations (Figure 2A, Table S4), Again, although the trend was present, this was not significant when the *p*-value was adjusted for false discovery. However, we did observe a statistically significant correlation between the total administrations of oxazolidinone and the change in Shannon diversity from BL to EOS (R = −0.581, *p* < 0.001, adj = 0.006), where those who had greater losses in Shannon diversity had greater total administrations of oxazolidinones (Figure 2B, Table S5). This finding suggests that higher administrations of oxazolidinones in patients who do not become colonized with CDC AR-threats likely reflects loss of unique ARGs able to potentiate ARC, which is a direct reflection of loss of total bacterial diversity.

### 2.2. The Impact of the Number of Unique Antibiotics on Patient Outcomes, ARGs, and Diversity

**Figure 3:**
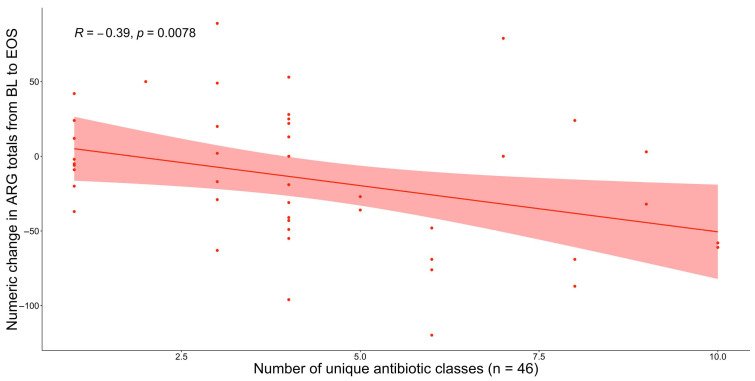
The numeric change in unique ARGs from BL to EOS vs. the unique number of antibiotic classes a patient receives. The scatterplot shows that the number of unique antibiotic classes received was negatively correlated with the numeric change in unique ARGs from BL to EOS among the 46 patients who had samples from both timepoints available. The correlation coefficients (R) and *p* values are derived from Spearman’s correlation rank test.

Next, we considered whether the number of unique antibacterials a patient receives influences their AR-threat outcomes. Interestingly, we did not find any significant differences in the number of unique antibiotic classes received between AR-threat outcome groups (non-ARI vs. ARI, non-ARC vs. ARC, no AR-threat vs. Any AR-threat) (Table S6), between patients who gained or lost ARGs from BL to EOS (Table S7), or amongst patients who gained or lost α-diversity (Table S8). However, we discovered a negative correlation between the number of unique antibiotic classes a patient received and the numeric change in unique ARGs mapping to a specific class from BL to EOS (Rs = −0.39, *p* = 0.008) (Figure 3). This is again likely a direct reflection of the loss of microbial diversity.

### 2.3. Antibiotic Exposure and AR-Threat or ARG Acquisition Risk

**Figure 4:**
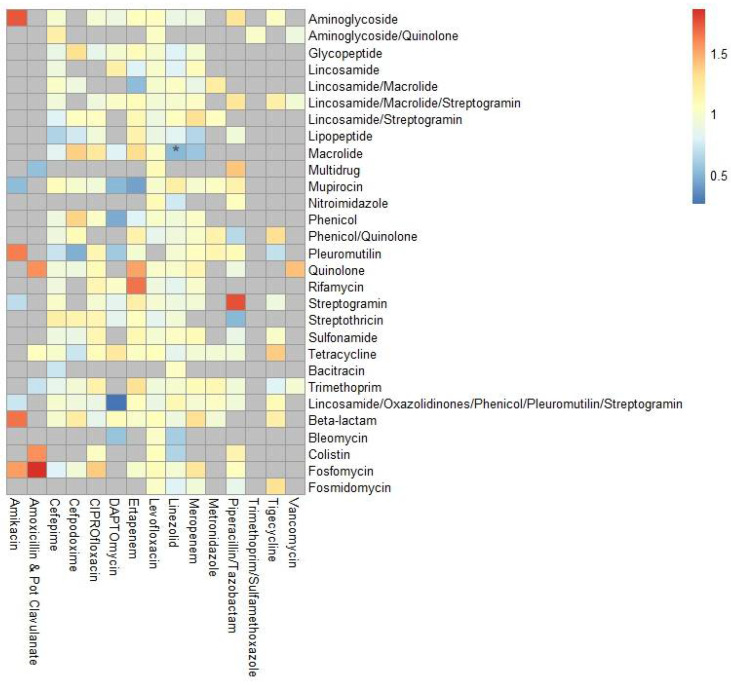
Odds of developing or acquiring an ARG from a particular antibiotic-resistance class in relation to antimicrobial exposure. The heatmap displays the odds ratios derived from GLMs assessing the relationship between the number of days of antibiotic exposure and the acquisition of antibiotic resistance genes across different antibiotics. Odds ratios were calculated from the model estimates and are represented by the color gradient, where red indicates increased odds, blue indicates decreased odds, and yellow represents neutral odds. Gray boxes indicate models that could not be computed due to insufficient data. Stars represent statistically significant associations where * *p* < 0.05.

Given that even within a specific class of drugs, there are slight differences in the spectrum of activity, we next analyzed time-varying antibiotic exposure of individual antibiotics as predictors of ARI, ARC, and any AR-threat event, assessing the risk for AR-threat outcomes with each additional day of antimicrobial exposure (Table 2 and Table S9). When treated as a time-varying covariate, each additional day of minocycline increased the risk of any AR-threat event by 170% (HR: 2.736, CI: 1.333–5.614, *p* = 0.006). On the other hand, each additional day of linezolid reduced the risk of ARC by ~30% (HR: 0.663, CI: 0.364–0.992, *p* = 0.046). Next, we ascertained if the odds of gaining specific ARG classes between baseline and the end of study were associated with cumulative days of antibiotic exposure to specific antibiotics (Figure 4, Tables S10 and S11). Linezolid decreased the odds of gaining genes predicted to confer macrolide resistance by ~50% (HR: 0.50, CI: 0.273–0.92, *p* = 0.026).

## 3. Discussion

The empirical use of broad-spectrum antibiotics for neutropenic fever is recommended as a standard of care among AML patients receiving IC to improve the survival rate among patients with suspected infection during cancer therapy [29]. However, the frequent use of antibiotics is known to exert selective pressure and drive the evolution of antibiotic resistance in bacterial populations [30]. Understanding the variables associated with antibiotic resistance is critical for designing appropriate interventions, improving patient outcomes, and minimizing the healthcare burden [31].

Data from the CDC’s 2019 AR Threats Report indicate that VRE, MRSA, ESBL, CRE, and MDRP infections are “Serious Threats” [12]. In 2019, these infections caused a combined total of 621,300 cases in the United States, resulting in an estimated 28,900 deaths [12]. These etiological agents are also the most frequent cause of infection in AML patients [32]. In addition to surveilling infections, monitoring gut colonization with bacteria that possess ARGs is particularly crucial for AML patients, as these patients are at a high risk of developing antibiotic-resistant infections caused by bacteria residing in the gut. Moreover, it is critical to monitor colonization with ARGs for potential transmission events in inpatient settings [33,34]. A study by Martinez-Nadal found that ~40% of neutropenic patients with bacteremia had inappropriate empirical antibiotic treatment. The highest risk for the improper choice of empirical therapy in neutropenic patients was bacteremia due to *S. maltophilia*, VRE, MDRP, coagulase-negative staphylococci, and ESBL *E. coli* [35]. Moreover, inappropriate therapy has been shown to increase mortality in neutropenic patients [36]. These findings have highlighted the need to assess the risk of infections with multidrug-resistant pathogens in cancer patients with febrile neutropenia to understand proper treatment choices.

Our lab has previously found that patients who lost microbial diversity over the course of RIC were significantly more likely to contract a microbiologically documented infection, and loss of diversity was linked to specific antimicrobial receipt [27]. Moreover, we also found that total days on antibiotics were significantly associated with increased temporal variability of the microbiome, where increased intra-patient temporal variability was correlated with increased risk of infection during RIC [28]. Herein, we aimed to take this a step further by integrating shotgun metagenomic sequencing data to assess the resistome as well as AR-threat events specifically. We first focused on whether the number of administrations (single doses) of specific antibiotic classes has any impact on AR-threat outcomes, including colonization and infection with CDC AR-threat pathogens, changes in the resistome, and/or microbiome diversity. The average number of oxazolidinone administrations was higher in AML patients who were not colonized by AR-threat pathogens (Figure 1). Although each additional day of linezolid administration decreased the odds of ARC (Table 2), our results did, however, show oxazolidinone treatment was associated with a noticeable reduction in bacterial diversity in AML patients over time (Figure 2). Our data also demonstrated that patients who received a greater number of unique antibiotics have a concomitant loss of unique ARG classes (Figure 3), which is likely a logical consequence of the loss of species carrying those unique ARGs. Although our previous studies suggest that loss of α-diversity is associated with subsequent infectious events [27,37], this data would suggest that antibiotic-induced loss of microbiome is not necessarily associated with AR-threat events, likely because of the loss of resistome diversity. One postulation might be, as it is known that AML patients are at higher risk of being infected with their own indigenous microflora [38], there is less opportunity for homologous recombination (i.e., transfer of MGEs or ARGs) to occur amongst organisms within the microbiome once the microbiome and ARG diversity is reduced. Thus, it is probable that a drug like linezolid, which is active against all clinically important Gram-positive bacteria as well as gut anaerobes such as *Clostridioides* spp., could vastly reduce the gut microbial diversity of AML patients, which are Firmicutes dominant during neutropenia [39], but also be negatively correlated with AR-events due to the “protection” against VRE and MRSA, while simultaneously maintaining an environment that is not conducive for ESBLs or CREs because they have no way to acquire those genes or no selection to mutate.

A previous study indicated that exposure to each additional day of antibiotic therapy is associated with an elevated risk of adverse effects and antibiotic-related harm [40]. Therefore, we evaluated whether exposure to individual antibiotics could indicate increased or reduced risk for ARI and/or ARC with CDC AR threats [12]. Again, each additional day of linezolid decreased the risk of ARC by 30% in a time-varying Cox model (Table 2). Moreover, using generalized linear models, our analysis showed that cumulative exposure to oxazolidinones decreased the odds of the acquisition of genes associated with resistance to macrolides (Figure 4). Given the spectrum of activity of linezolid is clinically relevant for Gram-positives such as *Enterococcus faecium*, *Staphylococcus aureus*, *Staphylococcus epidermidis*, and viridans group streptococci, linezolid is likely reducing the burden on these bacteria, which would also carry macrolide resistance genes [41,42,43]. Interestingly, our results are contrary to other publications, which have shown that treatment with antibiotics is correlated with increased ARG diversity and resistome features in hematologic malignancy patients [44]. However, to our knowledge, these studies analyze the correlation at a specific cross-sectional timepoint post-antibiotic treatment, without taking into consideration the baseline ARGs already present prior to antibiotic therapy, as we have in our GLM. So, although these studies show a correlation between specific antibiotics and grouped resistome features, it is possible these features were already present at baseline prior to treatment and thus selected for and not acquired. This is important to take into consideration, as it was previously shown that the baseline plasmidome is one of the major players in determining how an antibiotic would impact a patient’s resistome [45]. The benefit of our analyses reflects the odds of actual gene acquisition according to resistance to specific classes of antibiotics. Our results are more in line with other studies [46], which have observed no short-term enrichment of ARGs targeted to the specific antibiotics utilized in individuals, and most antibiotic exposures are associated with a decrease in the number of new ARGs [34]. As our patients are newly diagnosed treatment naïve patients, the differences in our study could also very well be due to short-term versus long-term consequences on the resistome after repeated exposure.

Our study has some limitations that should be considered. While we looked at the impact of antibiotics on ARG expansion, colonization by resistant pathogens, and the development of AR infections, the study’s retrospective design limits our ability to track previous antimicrobial exposure and long-term consequences of ARG expansion and the development of AR infections. We only know the antimicrobial exposures while on study, but do not know previous exposure before inpatient therapy. Moreover, due to the complex nature of frequent combinations or sequential antimicrobial exposure in these patients, this study does not account for the multifaceted nature of antimicrobial exposure. To our knowledge, few publications have explored synergistic antimicrobial effects on the microbiome or resistome [34,44,47]. Synergistic antimicrobial effects modeled for bactericidal, or AMR evolution effects have typically only considered antimicrobial treatment combinations, not modeling multiple antibiotics received at once, as seen in our patients. Moreover, we realize the number of AR-events and specific antimicrobial administrations within each group, as well as the single-center nature of our findings, limits the power of our analyses. Future studies with larger multi-institutional cohorts, longer follow-ups, and improved modeling of antimicrobial synergistic effects on the microbiota, as well as the evolution of antimicrobial resistance, would be beneficial for understanding the lasting impact of antibiotic use.

## 4. Materials and Methods

### 4.1. Study Design, Patients, and Sample Collection

Stool samples and longitudinal data were gathered from two different previously published/collected cohorts of newly diagnosed AML patients (n = 161) starting RIC at MD Anderson Cancer Center (MDACC) in Houston, TX, USA, from September 2013 to November 2019. One study focused on the microbiome and infectious toxicities during the induction phase (PA13-0339) [37], while the second studied infectious complications from baseline induction through consolidative therapy (PA15-0780). The study protocols were approved by the MDACC Institutional Review Board and were conducted in compliance with the Declaration of Helsinki. Written informed consent was obtained from all participants before enrollment. The 16S rRNA sequences of PA13-0339 were previously deposited in the NCBI Sequence Read Archive under the Bioproject IDs PRJNA352060 and PRJNA526551, while PA15-0780 cohort 16S rRNA sequences were previously deposited in the NCBI Sequence Read Archive under the Bioproject ID PRJNA1124986.

Briefly, the PA13-0339 cohort had stool samples collected (fresh frozen at −80 °C) from patients starting at baseline (BL) before RIC initiation, then approximately every 96 h as available, and was stopped upon neutrophil recovery as previously described [23,24]. These patients were followed for microbiologically documented infections (MDIs) per IDSA guidelines while on study and for 90 days post-neutrophil recovery as previously described [5,37]. The PA15-0780 cohort had stool samples collected at BL prior to RIC initiation, twice weekly for the first four weeks, once a week for weeks 4–8, every other week through weeks 8–12, and every two weeks up to 12–24 weeks, or loss to follow-up. These patients were followed for MDIs during the entire stool sampling window [5]. All patients from both cohorts were inpatients for RIC until neutrophil recovery and had fresh stools collected. After neutrophil recovery, outpatient samples were collected through rectal swabs, or if possible, some patients who followed post-inpatient stay were able to have fresh frozen stool samples collected at clinic follow-ups. Baseline samples were collected within a median time of 24 h of the first dose of IC. End-of-study samples (EOS) were collected a median of 28 days post-RIC initiation. Although PA15-0780 was designed to obtain more longitudinal sampling, sampling loss after the transition from inpatient to outpatient setting allowed the EOS sample time for most patients to be comparable. The time from BL to EOS was a median of 26 (IQR 18–75) days for the PA15-0780 cohort, versus 23 (IQR 20–28) days for PA13-0339. Patients who had available paired BL and EOS samples (n = 46) had a median time of 23 days from baseline to the end of sample.

Standard low-intensity regimens included low-dose cytarabine (LDAC) containing regimens, hypomethylating agents, or clinical trial treatments. Standard high-intensity chemotherapies included anthracyclines (daunorubicin or idarubicin), fludarabine, cytarabine combinations, or “7 + 3” equivalent regimens.

### 4.2. 16S Amplicon Sequencing and Identification of Potential Samples Colonized with Antibiotic-Resistant CDC-Threat Pathogens

The DNA was isolated from fecal samples by following the protocol from the QIA-amp DNA stool minikit (Qiagen) to include a bead-beating step for lysis [37]. The 16S rRNA gene V4 region was amplified by PCR from 100 ng of extracted genomic DNA using the 515F and 806R primer pair [29]. The amplicon libraries were sequenced using an Illumina MiSeq 2 × 250 bp paired-end protocol. The reads were demultiplexed, merged, and dereplicated for chimeras utilizing VSEARCH [30]. UNOISE3 was utilized for denoising and chimera calling [31]. Mothur was utilized to identify and taxonomically classify unique sequences against the SILVA Database (version 138). An operational taxonomic unit (OTU) table was generated using USEARCH, and α- and β- diversity metrics were determined using QIIME [31].

All longitudinal stool samples were analyzed for CDC AR-threat isolate extraction via 16S rRNA sequencing-guided selective culturing. All samples with a greater than 3% relative abundance of *Staphylococcus*, *Enterococcus*, Enterobacteriaceae, *Pseudomonas*, or *Klebsiella* were streaked directly onto HardyCHROM^TM^ CRE, HardyCHROM^TM^ ESBL, HardyCHROM^TM^ MRSA, or HardyCHROM^TM^ VRE agar (Santa Maria, CA, USA). Following isolation on antibiotic selective media, isolates were streaked on BBL Trypticase Soy Agar with 5% Sheep Blood (BD Biosciences, Milpitas, CA, USA) for single colony purification. The use of matrix-assisted laser desorption, ionization time-of-flight mass spectrometry (MALDI-TOF) was used to confirm the identification of these pure colonies to the species level (BD^TM^ Bruker MALDI Biotyper^®^, Billeria, MA, USA). Following species identification, antibiotic susceptibilities were determined using VITEK2 (bioMérieux, Marcy-l’Étoile, France). Gram-negative species were run on cards AST-GN69 and AST-XN06, while Gram-positive species were run on AST-GP75. If a patient had a stool sample found to have vancomycin-resistant *Enterococcus faecalis* or *Enterococcus faecium* (VRE), methicillin-resistant *Staphylococcus aureus* (MRSA), extended-spectrum beta-lactamase-producing or multidrug-resistant Enterobacteriaceae (ESBL), carbapenem-resistant Enterobacteriaceae (CRE), or multidrug-resistant *Pseudomonas aeruginosa* (MDRP), they were classified as having colonization with an AR CDC threat (ARC). The culture confirmed isolation of an AR-threat microorganism from the stool hospital surveillance swabs was also deemed an ARC.

### 4.3. Determination of Infection with an Antibiotic-Resistant CDC-Threat Pathogen

All MDI isolates identified by the clinical microbiology lab at any point while patients were on the study (between BL and EOS dates) were collected from the MD Anderson clinical microbiology laboratory and stored at –80 °C. The antibiotic susceptibility data of these isolates was determined by the clinical microbiology laboratory and were collected from the electronic medical record. Enzyme testing results (i.e., carbapenemase and beta-lactamase) were also collected. A patient was defined as having an infection with an AR CDC threat pathogen (ARI) if the patient contracted an infection caused by VRE, MRSA, ESBL-producing or multidrug-resistant Enterobacteriaceae, CRE, or MDRP. A patient was deemed to have “any AR-threat event” if they had either ARI or ARC. From baseline, the average time to ARI was approximately 29 days while the average time to ARC was 49 days.

### 4.4. Whole Genome Sequencing of AR-Colonization and AR-Infection Bacterial Isolates

Following the species identification of the bacterial isolates and their antibiotic susceptibilities, DNA was extracted from the isolates using the MasterPure Gram-positive DNA purification kit (Lucigen, Middleton, WI, USA). Libraries for DNA extracted from each isolate were prepared using the Illumina DNA Tagmentation Library Prep kit (Illumina, San Diego, CA, USA). The quality of the DNA library was assessed using the Qubit bioanalyzer and the Qubit dsDNA HS Assay Kit (Invitrogen, Waltham, MA, USA). Libraries were pooled and submitted to the North Texas Genome Center for sequencing on the Illumina NovaSeq 6000 S4 flow cell (Illumina, San Diego, CA, USA). An analysis of sequencing data was performed on the Grace computing cluster at Texas A&M University. Bacterial isolate data was down sampled to 6 million read pairs per sample using Seqtk (v1.3) and assembled in SPAdes (v3.14.1) under the “isolate” setting. Isolate assemblies were annotated on BV-BRC (https://www.patricbrc.org/) using the RAST toolkit-based pipeline. ARC and ARI bacterial isolates were deposited in the NCBI Sequence Read Archive under the Bioproject ID PRJNA1129516.

### 4.5. Shotgun Metagenomic Sequencing of Baseline and End of Study Samples

Fecal DNA was extracted from feces using a modified Qiagen Blood and Tissue kit (Qiagen, Valencia, CA, USA). Briefly, 150 mg of frozen feces was suspended in 500 µL of sterile InhibitEx Buffer (Qiagen, Valencia, CA, USA). Homogenization was accomplished by adding 150 mg of silicon beads (Lysing Matrix B, MP Biomedical, Santa Ana, CA, USA) and Hard Tissue Grinding Mix 2.4 mm metal beads (VWR, Radnor, PA, USA), and beating for 4 min at 60 s intervals at 4.5 m/s (MP Biomedical FastPrep-24 Classic). The suspension was heated for 7 min at 95 °C at 750 rpm. The homogenized mixture was centrifuged at 15,000 rpm for 3 min. Bacterial DNA was extracted from the supernatant, and the manufacturer’s instructions were followed by adding 20 µL of Proteinase K and Buffer AL, then vortexing. This mixture was then incubated at 70 °C for 30 min before 200 µL of 100% ethanol was added. This mixture was gently inverted several times before being transferred to a DNeasy spin column, where the manufacturer’s instructions for DNA cleanup were followed. The isolated DNA was stored at −20 °C.

Libraries for shotgun metagenomic samples were prepared for each sample using the Illumina Nextera XT DNA Library Prep kit (Illumina, San Diego, CA, USA). The quality of the DNA library was assessed using the Qubit bioanalyzer and the Qubit dsDNA HS Assay Kit (Invitrogen, Waltham, MA, USA). Libraries were pooled and submitted to the North Texas Genome Center for Illumina NovaSeq 6000 S4 sequencing (Illumina, San Diego, CA, USA) as above. Shotgun metagenomic samples were assembled using metaSPAdes (v3.14.1). The relative abundance of bacterial taxa in shotgun metagenomic samples was estimated using MetaPhlAn2 (v2.8.1). MetaSPAdes shotgun metagenomic assemblies were binned and annotated on PATRIC using the RAST Binning Service (RBS).

A total of 168 stool samples were included in the metagenomic analyses, including 98 BL samples and 70 EOS samples, representing 119 patients of the original 161 patients. The shortage of sample availability was a result of several factors, including unavailability of a true baseline (i.e., no availability of stool until well after initiation of chemotherapy), some patients did not complete the study, some patients were missing important metadata (i.e., antibiotic administration, infection outcomes, etc.), and some sample’s DNA and/or shotgun metagenomic sequencing did not pass quality standards. The PA13-0339 and PA15-0780 cohort shotgun metagenomic sequences were deposited in the NCBI Sequence Read Archive under the Bioproject IDs PRJNA1129514 and PRJNA1128111, respectively.

### 4.6. Resistome Analyses

Raw reads underwent quality control with fastp followed by assembly with Megahit (v1.2.8) for the BugSeq (v4.0) pipeline. Assemblies were taxonomically binned by BugSeq (v4.0), as previously described [32,33,34]. In brief, contigs were aligned against a curated reference sequence database using minimap2, and alignments were parsed for query coverage and absolute nucleotide identity (ANI) against reference sequences; these factors were combined to yield taxonomic classifications for each contig based on established ANI and coverage thresholds. Taxonomic bins were searched for genomic predictors of antimicrobial resistance (AMR) using BugSeq’s AMR analysis (v4.0). In brief, contigs were searched for proteins from a curated database containing over 6500 protein sequences associated with AMR. Protein search was performed with threshold alignment and Hidden Markov Model to accurately identify both gene allele and family. Taxon-specific models for phenotypic AMR prediction, which include single-nucleotide variants and other predictors of resistance (e.g., indels), were applied for ~50 clinically relevant bacterial species included in this proprietary pipeline. Taxonomic binning data and AMR predictions were combined into reports for downstream analysis.

### 4.7. Antibiotic Prescribing Practices and Antibiotic Administration Analyses

For newly diagnosed AML patients receiving RIC at MD Anderson Cancer Center, levofloxacin is typically used for prophylaxis. If it cannot be used for reasons such as intolerance or drug–drug interactions, then cefpodoxime is used. For unspecific neutropenic fever, cefepime is typically the first-line empirical therapy of choice in the event of neutropenic fever. Piperacillin-tazobactam is utilized if an anaerobic infection is considered likely. Meropenem is generally used when first-line agents do not resolve the fever, the patient is in septic shock, or a drug-resistant bacterium is identified. Linezolid is typically given in combination with cefepime (or the other agents noted above) to cover resistant Gram-positive organisms (MRSA, VRE, viridians group streptococci, etc.) while cultures are pending. If none of these organisms are identified, then linezolid should be stopped after 48–72 h unless the patient has pneumonia or skin/soft tissue infection where cultures might not be positive.

All antibiotic use for each patient from the time of BL to EOS was extracted from a database maintained by pharmacy informatics as previously described [35]. Antibiotic use was assessed at the individual drug level and considered as both total administration (i.e., any dose) and cumulative use (i.e., total days of therapy during the study period). An antimicrobial therapy day was defined as any single calendar day (24 h) on which an antibiotic was administered, regardless of dose or dosing frequency. Only antibacterials were considered. We accounted for 3 routes of administration (intravenous, oral, and injection), other routes of administration were not included, given that less than 1% of patients received them, and thus, it was likely that analyses would not be statistically powered. Several antibiotics had too few patients in the comparison groups and thus are denoted as “NAs”. To understand how antibiotic administration factors could be associated with AR-threat events, antibiotic administration data were only considered up to the time of the first AR-event (ARI, ARC, or the first AR-threat event if patients had both). If the patient did not experience any of those events, only up to 8 weeks (56 days) of data or the end of the study, whichever came first, was incorporated. For consideration of the impact of antibiotic administration on microbial diversity or ARGs, administration was considered between BL to EOS.

### 4.8. Statistical Analyses

To visually confirm normality, we used QQ-plots and density plots (R package: ggpubr), in addition to running the Shapiro–Wilk test. The Mann–Whitney U test was used for non-parametric 2-group comparisons. Spearman’s rank correlation was used to test the strength and direction of the association between discrete variables while the point biserial correlation test was used for the association between binary and discrete variables. The effect of antibiotic use on the emergence of ARI or ARC in patients was analyzed with a time-varying Cox proportional hazards model (R packages: survival, and survsim). In which antibiotic use was a time-dependent variable used to estimate hazard ratios and their corresponding 95% confidence intervals. Antibiotic exposure was considered on a day-by-day basis, accounting for periods both on and off antibiotics throughout the study period. ARI, ARC, or both were independently tested as the binary variable. Antibiotic administration and resistome data of patients were included up to the date of infection or colonization, while patients who experienced no event only considered data up to 56 days. After determining that the average number of days from the start of chemotherapy to the event of AR-colonization was 49 days, the cutoff of 56 days was chosen to have the most similar number of days studied for all patients. For patients who had multiple ARI or ARC isolates collected, only the first isolate/event was included in time-to-event analyses. The hazard ratios presented are unadjusted, as no clinical variables met criteria for confounding and thus no adjustment was performed. Each antibiotic was analyzed separately, where each analysis only included only patients who received that specific antibiotic. Additionally, we examined the effect of antibiotic use on the gain of specific ARG classes or loss of Shannon diversity from BL to EOS in patients. Binary logistic regression models were used to evaluate the association between ARG gain or Shannon diversity loss and predictor variables. The model included two fixed effects: the duration of antibiotic use (Antibiotic_days) and baseline ARG counts. A generalized linear model (GLM) was implemented with a binomial distribution and logit link function to account for the binary outcome variables (gain of ARGs). The analysis was conducted using the following formula:

Model = glm (ARG_gain ~ Antibiotic_days + baseline_ARG_counts, family  = binomial (link = “logit”), data = data)

The dataset (data) contained a single measure of ARG gain from baseline to end of study, with no repeated measures or random intercepts for individual patients, as repeated measures were not applicable. The logistic models were adjusted for the number of baseline ARGs only, with no additional clinical variables included. Each model included only patients who received the specific antibiotic. All tests were conducted using the R version (4.3.0). Statistical significance was defined as *p*-values less than 0.05.

## 5. Conclusions

Overall, these findings provide valuable observations into the specific antimicrobial effects on resistome diversity and AR-events during IC. Most importantly, while specific antibiotics may reduce microbial diversity, they are not necessarily linked to the expansion of ARGs or AR-threat events. By further understanding additional risk factors, healthcare providers can more effectively tailor antibiotic regimens, preserving the efficacy of future treatments, and minimizing microbial dysbiosis-related complications.

## Figures and Tables

**Table 1:** Characteristics of patients used in this study.

Patient Characteristics	Total	Paired ^a^
Patient count	119	46
Sex, N (%)		
Female	56 (47.06)	18 (39.13)
Male	57 (47.9)	25 (54.35)
Unavailable information	6 (5.04)	3 (6.52)
Chemotherapy intensity, N (%)		
High	66 (55.46)	19 (41.3)
Low	47 (39.5)	24 (52.17)
Unavailable information	6 (5.04)	3 (6.52)
No. of patients with confirmed ARI, N (%) ^b^	8 (6.72)	4 (8.7)
No. of ARI isolates	12	6
*Pseudomonas aeruginosa*	6	2
*Staphylococcus aureus*	1	0
*Escherichia coli*	5	4
No. of patients with confirmed ARC, N (%) ^c^	14 (11.76)	7 (15.22)
No. of ARC isolates	18	9
*Enterobacter cloacae*	1	1
*Klebsiella pneumoniae*	3	1
*Pseudomonas aeruginosa*	1	1
*Enterococcus faecium*	6	2
*Escherichia coli*	3	2
*Staphylococcus aureus*	4	2
Antibiotic Administration, N (Total Administrations)	BL to Event or 56 days	BL to EOS
Aminoglycosides	22 (59)	10 (26)
Carbapenems	59 (1299)	22 (650)
Cephalosporins	88 (2088)	31 (804)
Fluoroquinolones	101 (1318)	41 (663)
Glycopeptide	7 (89)	5 (147)
Lincosamides	4 (63)	0 (0)
Lipopeptide	35 (199)	13 (71)
Macrolides	6 (37)	2 (17)
Monobactam	12 (194)	4 (37)
Nitroimidazole	22 (296)	8 (118)
Oxazolidinone	90 (1181)	32 (473)
Penicillins	39 (835)	16 (213)
Sulfonamides	11 (180)	3 (105)
Tetracyclines	30 (372)	13 (103)

^a^ Refers to the patients who had paired BL and EOS samples available for analyses. ^b^ 4 patients had single ARI isolates, while 4 had multiple ARI isolates collected. ^c^ 7 patients had single ARC isolates, while 7 had multiple ARC isolates collected.

**Table 2:** Hazards of experiencing an AR-event from antimicrobial usage.

	Hazard Ratios, Confidence Intervals, and *p*-Values ^a^		
Antibiotic	ARI	ARC	Any AR-Threat Event
Amikacin	0.937 (0.324–2.707) [0.905]	0.933 (0.272–3.207) [0.913]	0.634 (0.193–2.081) [0.452]
Amoxicillin and clavulanate	NA ^b^	0.375 (0.119–1.171) [0.091]	0.272 (0.072–1.024) [0.054]
Azithromycin	NA	NA	0.6841 (0.37–1.973) [0.483]
Cefepime HCL	0.997 (0.499–1.994) [0.994]	0.687 (0.272–1.73) [0.425]	0.846 (0.449–1.593) [0.604]
Cefpodoxime	0.890 (0.385–2.056) [0.785]	1.256 (0.608–2.597) [0.538]	1.305 (0.729–2.330) [0.37]
Ciprofloxacin	0.718 (0.206–2.508) [0.603]	0.573 (0.163–2.011) [0.384]	0.466 (0.157–1.376) [0.167]
Daptomycin	1.315 (0.431–4.011) [0.631]	0.510 (0.120–1.857) [0.307]	0.923 (0.373–2.280) [0.862]
Ertapenem Sodium	0.657 (0.115–3.768) [0.638]	NA	0.596 (0.101–3.504) [0.567]
Levofloxacin	0.832 (0.392–1.766) [0.631]	0.719 (0.450–1.148) [0.167]	0.719 (0.443–1.166) [0.181]
Linezolid	0.565 (0.214–1.491) [0.249]	0.663 (0.364–0.992) [0.047] *	0.662 (0.402–1.09) [0.105]
Meropenem	3.411 (0.0753–1.141) [0.077]	0.881 (0.436–1.777) [0.722]	0.640 (0.291–1.405) [0.266]
Metronidazole	0.523 (0.239–1.144) [0.104]	1.563 (0.912–2.677) [0.104]	0.912 (0.440–1.89) [0.805]
Minocycline	NA	1.229 (0.368–4.108) [0.737]	2.736 (1.333–5.614) [0.006] *
Piperacillin/Tazobactam	1.022 (0.439–2.377) [0.959]	0.883 (0.311–2.51) [0.816]	1.211 (0.607–2.414) [0.587]
Tigecycline	1.295 (0.391–4.285) [0.672]	NA	1.413 (0.588–3.397) [0.440]

^a^ Hazard ratios (HR), *p*-values, and confidence intervals refer to hazards associated with each additional day of antibiotic exposure (cumulative exposure); the first value is the hazard ratio with the confidence interval placed in brackets after the semi-colon; all values are calculated from Cox Proportional Hazards model. ^b^ If too few patients were available in each group to be statistically accurate, it was listed as NA. The antibiotics not listed had too few patients available in each group or too few administrations to be statistically accurate.* *p*-value < 0.05.

## Data Availability

Publicly available datasets were analyzed in this study. This data can be found at the NCBI Sequence Read Archive (https://www.ncbi.nlm.nih.gov/sra) under the BioProject IDs PRJNA352060, PRJNA526551, and PRJNA1124986.

## Supplementary Materials

The following supporting information can be downloaded at https://www.mdpi.com/article/10.3390/antibiotics14080770/s1, Table S1—Comparison of the total number of administrations for individual antimicrobials between AR-threat outcome groups; Table S2—Comparison of the total number of administrations of individual antibiotics between patients who either lost or gained unique antibiotic resistance genes from BL to EOS; Table S3—Correlation between the total number of administrations of individual antibiotics and the numeric change in ARGs between BL and EOS; Table S4—Comparisons of the total number of administrations of individual antibiotics between patients who either lost or gained Shannon diversity between BL and EOS; Table S5—Correlation between the total number of administrations of individual antibiotics and the numeric change in Shannon diversity between BL and EOS; Table S6—Comparisons of the unique number of antibiotics a patient received among patient AR-threat outcome groups; Table S7—Comparison of the unique number of antibiotics a patient received among patients who gained or lost ARGs from BL to EOS; Table S8—Comparison of the unique number of antibiotics a patient received among patients who gained or lost Shannon diversity from BL to EOS; Table S9—Sample sizes for time-varying Cox proportional hazards models; Table S10—Sample Sizes for GLMs; Table S11—Odds of ARG acquisition associated with antimicrobial usage.

## Abbreviations

The following abbreviations are used in this manuscript:

AML	acute myeloid leukemia
AMR	antimicrobial resistance
AR	Antibiotic resistant
RIC	remission induction chemotherapy
ARI	antibiotic resistant infection
ARC	antibiotic resistant colonization
BL	baseline
EOS	end of study
ARG	antimicrobial resistance genes
CDC	Center for Disease Prevention
MDRP	multidrug resistant *Pseudomonas aeruginosa*
ESBL	extended-spectrum beta-lactamase producing Enterobacteriaceae
MRSA	methicillin-resistant *Staphylococcus aureus*
VRE	vancomycin-resistant *Enterococcus*
CRE	carbapenem-resistant Enterobacteriaceae
MGE	mobile genetic element
ANI	absolute nucleotide identity
NA	not applicable
CI	confidence interval
