# Supplementary material for: Characterization of Antibiotic Administration Factors Associated with Microbiome Disruption and Subsequent Antibiotic-Resistant Infection and Colonization Events in Acute Myeloid Leukemia Patients Receiving Chemotherapy
Source: Antibiotics (Basel). 2025 Jul 30;14(8):770. doi: 10.3390/antibiotics14080770 (PMC12382674; doi:10.3390/antibiotics14080770)
Supplement: Supplementary file 1 [file antibiotics-14-00770-s001.zip › antibiotics-3516241-supplementary.pdf]

Table S1 - Comparison of the total number of administrations for individual antimicrobial classes between AR-threat outcome groups.

Classes	N; Median Individual Administrations (Range)[a]						P-values[b]			Adjusted p-values[c]		
	Non-ARI	ARI	Non-ARC	ARC	No-AR threat event	Any AR-threat event	Non-ARI vs. ARI	Non-ARC vs. ARC	No AR-threat event vs. Any AR-threat event	Non-ARI vs. ARI	Non-ARC vs. ARC	No AR-threat event vs. Any AR-threat event
Aminoglycosides	19; 2 (1-8)	3; 2 (1-7)	20; 2 (1-8)	2; 4 (1-7)	18; 2 (1-8)	4; 2 (1-7)	0.392	0.774	0.783	0.948	1	1
Carbapenems	55; 14 (1-99)	4; 7 (2-24)	54; 11 (1-99)	5; 27 (17-40)	51; 13 (1-99)	8; 17 (2-40)	0.242	0.020*	0.562	0.847	0.222	0.993
Cephalosporins	81; 11 (1-71)	7; 10 (1-32)	79; 11 (1-71)	9; 16 (1-55)	74; 11 (1-71)	14; 10 (1-55)	0.155	0.85	0.497	0.726	1	0.993
Fluoroquinolones	94; 9 (1-46)	7; 13 (2-27)	89; 9 (1-46)	12; 11 (1-27)	85; 9 (1-46)	16; 10 (1-27)	0.513	0.891	0.628	1	1	0.993
Glycopeptide	6; 6 (1-57)	1; 3 (3-3)	6; 6 (1-57)	1; 2 (2-2)	5; 9 (1-57)	2; 2 (2-3)	1	0.801	0.845	1	1	1
Lincosamides	4; 14 (4-32)	0	2; 18 (4-32)	2; 14 (12-15)	2; 18 (4-32)	2; 14 (12-15)	NA	1	1	NA	1	1
Lipopeptide	32; 5 (1-14)	3; 6 (1-12)	32; 5 (1-14)	3; 4 (3-4)	29; 5 (1-14)	6; 4 (1-12)	0.859	0.357	0.597	1	1	0.993
Macrolides	6; 6 (3-11)	0	4; 6 (3-7)	2; 8 (5-11)	4; 6 (3-7)	2; 8 (5-11)	NA	0.639	0.639	NA	1	0.993

Monobactam	12; 9 (1-70)	0	10; 10 (2-70)	2; 3 (1-5)	10; 10 (2-70)	2; 3 (1-5)	NA	0.067	0.067	NA	0.234	0.468
Nitroimidazole	19; 11 (1-68)	3; 2 (1-10)	19; 10 (1-68)	3; 11 (2-26)	17; 11 (1-68)	5; 10 (1-26)	0.076	0.962	0.479	0.678	1	0.993
Oxazolidinones	84; 12 (1-51)	6; 4 (1-24)	79; 12 (1-51)	11; 4 (3-28)	75; 12 (1-51)	15; 4 (1-28)	0.097	0.032*	0.024*	0.678	0.222	0.338
Penicillins	35; 10 (1-52)	4; 13 (10-33)	32; 14 (1-49)	7; 10 (3-52)	30; 14 (1-49)	9; 10 (3-52)	0.406	0.451	1	0.948	1	1
Sulfonamides	11; 7 (1-84)	0	11; 7 (1-84)	0	11; 7 (1-84)	0	NA	NA	NA	NA	NA	NA
Tetracyclines	26; 8 (1-58)	4; 6 (1-30)	27; 8 (1-58)	3; 16 (11-30)	24; 8 (1-58)	6; 11 (1-30)	0.876	0.047*	0.381	1	0.222	0.993

[a] First value represents the sample size within the corresponding outcome group of patients receiving that particular antimicrobial; the value followed by the semi-colon is the mean total administrations of the drug within that group; with the range of total administrations in parentheses.

[b] Resulting p-values calculated from the Mann-Whitney U Test

[c] Adjusted p-values derived from Benjamini-Hochberg method

* P value < 0.05

Table S2 - Comparison of the total number of administrations of individual antibiotic classes between patients who either lost or gained unique antibiotic resistance genes from BL to EOS.

**Classes (n_L, n_G)[a]**	**Median Number of Administrations (Range)[b]**		**P-value[c]**	**Adjusted p-value[d]**
	**Loss (L)**	**Gain (G)**		
Aminoglycosides (n_L = 6, n_G = 3)	2 (1-3)	1 (1-7)	1	1
Carbapenems (n_L = 16, n_G = 4)	30 (3-95)	28 (11-47)	0.925	1
Cephalosporins (n_L = 20, n_G = 11)	20 (2-79)	16 (4-64)	0.62	1
Fluoroquinolones (n_L = 24, n_G = 15)	14 (3-32)	13 (1-29)	0.386	1
Glycopeptide (n_L = 4, n_G = 0)	34 (1-57)	NA	NA	NA
Lipopeptide (n_L = 6, n_G = 7)	4 (1-14)	5 (1-14)	0.829	1
Macrolides (n_L = 2, n_G = 0)	8 (1-6)	NA	NA	NA
Monobactam (n_L = 2, n_G = 2)	11 (8-14)	8 (5-10)	0.667	1
Nitroimidazole (n_L = 6, n_G = 1)	4 (1-30)	12 (12-12)	0.801	1
Oxazolidinones (n_L = 21, n_G = 9)	16 (4-49)	7 (1-17)	0.03*	0.384
Penicillins (n_L = 9, n_G = 5)	14 (1-23)	8 (1-33)	1	1
Sulfonamides (n_L = 2, n_G = 1)	41 (33-49)	23 (23-23)	0.667	1
Tetracyclines (n_L = 10, n_G = 3)	4 (1-19)	8 (6-14)	0.235	1

[a] n_L represents the number of patients from the loss group that is administered that particular antibiotic; n_G represents the number of patients from the gain group that is administered that particular antibiotic)

[b] The median number of administrations for a particular antibiotic within that group; the range of the number of administrations for a particular antibiotic is in parentheses

[c] Resulting p-values calculated from Mann-Whitney U test

[d] Adjusted p-values derived from Benjamini-Hochberg method

* P value < 0.05

Note: Some antibiotics do not appear in the table due to too few patients in groups to be statistically powered. Patients that experienced neither a lost or gain ARGs from BL to EOS were not included in this analysis.

Table S3 – Correlation between the total number of administrations of individual antibiotics and the numeric change in ARGs between BL and EOS.

**Classes (n)**[a]	**Rho**[b]	**P-value**[c]	**Adjusted P-value**[d]
Aminoglycosides (n = 10)	0.039	0.915	1
Carbapenems (n = 22)	0.054	0.811	1
Cephalosporins (n = 31)	0.114	0.54	1
Fluoroquinolones (n = 41)	0.054	0.738	1
Glycopeptide (n = 5)	0.1	0.95	1
Lipopeptide (n = 13)	-0.083	0.788	1
Macrolides (n = 2)	1	1	1
Monobactam (n = 4)	-0.8	0.333	1
Nitroimidazole (n = 8)	0.307	0.459	1
Oxazolidinones (n = 32)	-0.327	0.067	0.876
Penicillins (n = 16)	0.121	0.655	1
Sulfonamides (n = 3)	-1	0.333	1
Tetracyclines (n = 13)	0.29	0.336	1

[a] n represents the number of patients receiving that particular antibiotic.

[b,c] Resulting rhos and p-values calculated from Spearman's correlation rank test.

[d] Adjusted p-values derived from Benjamini-Hochberg method

* P value < 0.05

Table S4 – Comparisons of the total number of administrations of individual antibiotics between patients who either lost or gained Shannon diversity between BL and EOS.

Classes (n_L, n_G)[a]	Median Number of Administrations (Range)[b]		P-value[c]	Adjusted p-value[d]
	Loss (L)	Gain (G)		
Aminoglycosides (n_L = 9, n_G = 1)	1 (1-7)	3 (3-3)	0.454	1
Carbapenems (n_L = 18, n_G = 4)	35 (3-95)	14 (11-22)	0.067	0.435
Cephalosporins (n_L = 20, n_G = 11)	22 (2-64)	16 (4-79)	0.385	1
Fluoroquinolones (n_L = 25, n_G = 16)	14 (3-46)	14 (1-30)	0.529	1
Glycopeptide (n_L = 5, n_G = 0)	21 (1-57)	NA	NA	NA
Lipopeptide (n_L = 10, n_G = 3)	4 (1-14)	5 (2-7)	0.932	1
Macrolides (n_L = 2, n_G = 0)	8 (1-16)	NA	NA	NA
Monobactam (n_L = 2, n_G = 2)	9 (8-10)	10 (5-14)	1	1
Nitroimidazole (n_L = 7, n_G = 1)	12 (1-40)	1 (1-1)	0.272	1
Oxazolidinones (n_L = 22, n_G = 10)	16 (1-49)	6 (3-49)	0.008*	0.1
Penicillins (n_L = 9, n_G = 7)	14 (1-23)	9 (1-33)	0.632	1
Sulfonamides (n_L = 3, n_G = 0)	33 (23-49)	NA	NA	NA
Tetracyclines (n_L = 10, n_G = 3)	6 (1-19)	5 (3-10)	1	1

[a] n_L represents the number of patients from the loss group that is administered that particular antibiotic; n_G represents the number of patients from the gain group that is administered that particular antibiotic

[b] The median number of administrations for a particular antibiotic within that group; the range of the number of administrations for a particular antibiotic is in parentheses

[c] Resulting p-values calculated from Mann-Whitney U test

[d] Adjusted p-values derived from Benjamini-Hochberg method

* P value < 0.05

Table S5 – Correlation between the total number of administrations of individual antibiotics and the numeric change in Shannon diversity between BL and EOS.

**Classes (n)**[a]	**Rho**[b]	**P-value**[c]	**Adjusted p-value**[d]
Aminoglycosides (n = 10)	0.488	0.152	0.66
Carbapenems (n = 22)	-0.218	0.329	0.712
Cephalosporins (n = 31)	-0.162	0.384	0.712
Fluoroquinolones (n = 41)	-0.018	0.912	1
Glycopeptide (n = 5)	0.1	0.95	1
Lipopeptide (n = 13)	0.481	0.096	0.623
Macrolides (n = 2)	1	1	1
Monobactam (n = 4)	0.2	0.917	1
Nitroimidazole (n = 8)	0.012	0.978	1
Oxazolidinones (n = 32)	-0.581	0.0005*	0.006*
Penicillins (n = 16)	0.121	0.655	1
Sulfonamides (n = 3)	-1	0.333	0.712
Tetracyclines (n = 13)	0.37	0.214	0.695

[a] n represents the number of patients receiving that particular antibiotic.

[b,c] Resulting rhos and p-values calculated from Spearman’s correlation rank test.

[d] Adjusted p-values derived from Benjamini-Hochberg method

* P value < 0.05

Table S6 – Comparisons of the unique number of antibiotic classes a patient received among patient AR-threat outcome groups.

**Comparisons (n)**[a]	**Median (Range)**[b]		**P-value**[c]	**Adjusted P-Value**[d]
	**Non-Event**	**Event**		
Non-ARI (n = 111) vs. ARI (n = 8)	4 (1-10)	4 (3-8)	0.312	0.609
Non-ARC (n = 105) vs. ARC (n = 14)	4 (1-10)	4 (1-7)	0.904	0.904
No AR-threat event (n = 100) vs. Any AR-threat event (n = 19)	4 (1-10)	5 (1-8)	0.406	0.609

[a] n represents the number of patients within the group

[b] The first value is the median number of unique antibiotics classes administered within that group; the range of the number of unique antibiotics administered is in parentheses

[c] Resulting p-values calculated from Mann-Whitney U test

[d] Adjusted p-values derived from Benjamini-Hochberg method

* P value < 0.05

Table S7 – Comparison of the unique number of antibiotics classes a patient received among patients who gained or lost ARGs from BL to EOS.

**Group (n)**[a]	**Median (Range)**[b]	**P-value**[c]
Lose (n = 28)	4 (1-10)	0.28
Gain (n = 16)	4 (1-9)	

[a] n represents the number of patients within the group

[b] The first value is the median number of unique antibiotics administered within that group; the range of the number of unique antibiotics administered is in parentheses

[c] Resulting p-values calculated from Mann-Whitney U test

Table S8 – Comparison of the unique number of antibiotics a patient received among patients who gained or lost Shannon diversity from BL to EOS

**Group (n)**[a]	**Median (Range)**[b]	**P-value**[c]
Lose (n = 29)	4 (1-10)	0.102
Gain (n = 17)	4 (1-6)	

[a] n represents the number of patients within the group

[b] The first value is the median number of unique antibiotics administered within that group; the range of the number of unique antibiotics administered is in parentheses

[c] Resulting p-values calculated from Mann-Whitney U test

Table S9. Sample sizes for time-varying Cox proportional hazards models

	**Patients experiencing the event, total patients in analysis**		
**Antibiotic**	**ARI**	**ARC**	**Any AR-threat event**
Amikacin	6,26	5,26	8,26
Amoxicillin & Pot Clav	NA	3,22	5,23
Azithromycin	NA	NA	3,8
Cefepime HCL	7,79	8,79	13,79
Cefpodoxime	4,38	6,38	9,38
Ciprofloxacin	6,32	6,32	10,32
Daptomycin	4,38	3,38	7,38
Ertapenem Sodium	5,28	NA	6,28
Levofloxacin	6,94	11,94	15,94
Linezolid	9,92	12,92	17,92
Meropenem	8,59	7,59	12,59
Metronidazole	4,24	3,24	6,24
Minocycline	NA	3,20	6,20
Piperacillin/Tazobactam	4,34	5,34	7,34
Tigecycline	4,21	NA	5,21

Table S10. Sample Sizes for GLM models

Antibiotic	Patients who received each antibiotic with paired BL and EOS samples available
Amikacin	8
Amoxicillin & Pot Clavulanate	5
Cefepime HCL	27
Cefpodoxime Proxetil	11
Ciprofloxacin	13
Daptomycin	13
Ertapenem Sodium	12
Levofloxacin	35
Linezolid	32
Meropenem	19
Metronidazole	8
Minocycline	6
Piperacillin/Tazobactam	14
Trimethoprim/Sulfamethoxazole	3
Tigecycline	9
Vancomycin	5

Table S11. Odds of ARG acquisition associated with antimicrobial usage.

**ARG** [a,b]	Amikacin	Amoxicillin	Cefepime	Cefpodoxime	CIPROfloxacin	DAPTOmycin	Ertapenem	Levofloxacin	Linezolid	Meropenem	Metronidazole	Piperacillin/ Tazobactam	Trimethoprim/ Sulfamethoxazole	Tigecycline	Vancomycin
AMINOGLYCOSIDE	1.735 (0.559-5.382) [0.340]		0.985 (0.834-1.162) [0.857]		0.972 (0.771-1.225) [0.811]	0.895 (0.659-1.217) [0.48]	1.066 (0.848-1.339) [0.585]	1.049 (0.952-1.155) [0.332]	0.891 (0.752-1.056) [0.184]	0.916 (0.758-1.106) [0.36]		1.271 (0.821-1.966) [0.282]		1.037 (0.521-2.065) [0.918]	
AMINOGLYCOSIDE/ QUINOLONE			1.173 (0.871-1.579) [0.293]					1.032 (0.814-1.308) [0.796]					1 (0.480-2.085) [1]		0.905 (0.597-1.371) [0.637]
GLYCOPEPTIDE			0.864 (0.677-1.102) [0.239]	1.296 (0.914-1.839) [0.146]	0.845 (0.641-1.116) [0.236]	0.959 (0.731-1.256) [0.759]	1.081 (0.864-1.351) [0.496]	0.989 (0.9-1.085) [0.808]	0.788 (0.587-1.058) [0.113]	0.968 (0.753-1.243) [0.797]					
LINCOSAMIDE			0.922 (0.725-1.173) [0.510]			1.18 (0.795-1.75) [0.411]	0.784 (0.447-1.374) [0.395]	0.999 (0.9-1.109) [0.985]	0.805 (0.592-1.093) [0.164]	1.072 (0.825-1.394) [0.601]					
LINCOSAMIDE/ MACROLIDE			1.018 (0.855-1.213) [0.839]	0.899 (0.667-1.213) [0.487]			0.513 (0.089-2.97) [0.456]	0.974 (0.872-1.088) [0.636]	1.022 (0.874-1.196) [0.784]	0.87 (0.646-1.173) [0.361]	1.202 (0.771-1.875) [0.417]				
LINCOSAMIDE/ MACROLIDE/ STREPTOGRAMIN			0.928 (0.755-1.142) [0.482]		0.924 (0.769-1.11) [0.398]	1.02 (0.691-1.505) [0.922]	1.072 (0.857-1.339) [0.543]	0.943 (0.836-1.062) [0.333]	0.974 (0.833-1.139) [0.743]	1.023 (0.88-1.189) [0.768]		1.253 (0.722-2.174) [0.422]		1.172 (0.706-1.946) [0.54]	0.944 (0.633-1.408) [0.779]
LINCOSAMIDE/ STREPTOGRAMIN			0.805 (0.528-1.226) [0.312]	1.044 (0.547-1.993) [0.896]	1.039 (0.848-1.272) [0.715]		1.109 (0.893-1.377) [0.349]	0.905 (0.773-1.058) [0.209]	1.052 (0.84-1.316) [0.66]	1.28 (0.9-1.821) [0.17]	1.039 (0.608-1.775) [0.89]				
LIPOPEPTIDE			0.622 (0.266-1.457) [0.275]	0.746 (0.372-1.497) [0.409]	0.928 (0.706-1.22) [0.592]		1.157 (0.916-1.461) [0.222]	0.885 (0.686-1.142) [0.348]	0.826 (0.569-1.198) [0.313]	0.646 (0.264-1.577) [0.337]		0.93 (0.448-1.933) [0.846]			
MACROLIDE			0.818 (0.604-1.109) [0.196]	1.372 (0.652-2.887) [0.405]	1.246 (0.849-1.827) [0.261]	0.806 (0.561-1.157) [0.242]	1.311 (0.755-2.276) [0.336]	1.008 (0.912-1.114) [0.877]	0.501 (0.273-0.92) [0.026]	0.562 (0.217-1.457) [0.236]					
MULTIDRUG		0.530 (0.0777-3.620) [0.517]						1.097 (0.922-1.307) [0.296]				1.402 (0.743-2.646) [0.297]			

**ARG** [a,b]	Amikacin	Amoxicillin	Cefepime	Cefpodoxime	CIPROfloxacin	DAPTOmycin	Ertapenem	Levofloxacin	Linezolid	Meropenem	Metronidazole	Piperacillin/ Tazobactam	Trimethoprim/ Sulfamethoxazole	Tigecycline	Vancomycin
NITROIMIDAZOLE								1.093 (0.86-1.389) [0.466]	0.739 (0.339-1.608) [0.445]			1.049 (0.554-1.984) [0.884]			
PHENICOL			0.912 (0.725-1.147) [0.430]	1.357 (0.928-1.983) [0.115]	1.013 (0.832-1.235) [0.895]	0.45 (0.067-3.013) [0.41]	0.794 (0.557-1.133) [0.204]	0.993 (0.883-1.117) [0.907]	0.928 (0.786-1.095) [0.375]	1.017 (0.812-1.273) [0.886]					
PHENICOL/ QUINOLONE			0.918 (0.645-1.306) [0.635]	1.086 (0.807-1.462) [0.586]			1.16 (0.877-1.533) [0.299]	0.853 (0.637-1.143) [0.288]	0.917 (0.708-1.187) [0.509]	0.991 (0.797-1.232) [0.933]	1.16 (0.739-1.82) [0.518]	0.654 (0.276-1.549) [0.335]		1.307 (0.594-2.874) [0.506]	
PLEUROMUTILIN	1.631 (0.475-5.605) [0.437]		0.703 (0.275-1.797) [0.461]	0.466 (0.044-4.892) [0.524]	1.12 (0.848-1.479) [0.426]	0.574 (0.112-2.944) [0.506]	0.963 (0.726-1.277) [0.791]		0.964 (0.712-1.303) [0.81]	1.044 (0.878-1.241) [0.625]	1.13 (0.798-1.601) [0.49]	1.091 (0.683-1.742) [0.716]		0.689 (0.204-2.325) [0.549]	
QUINOLONE		1.574 (0.611-4.055) [0.347]	0.933 (0.764-1.140) [0.497]	0.893 (0.632-1.262) [0.522]	1.089 (0.906-1.308) [0.364]		1.511 (0.936-2.442) [0.091]	0.993 (0.889-1.11) [0.904]	0.993 (0.856-1.152) [0.927]	1.129 (0.942-1.354) [0.19]		0.906 (0.617-1.33) [0.615]			1.409 (0.448-4.43) [0.558]
RIFAMYCIN			0.923 (0.703-1.212) [0.564]		1.131 (0.84-1.522) [0.419]	1.019 (0.764-1.359) [0.897]	1.649 (0.674-4.033) [0.273]	0.92 (0.806-1.051) [0.22]	0.84 (0.637-1.108) [0.218]	0.992 (0.8-1.229) [0.939]					
STREPTOGRAMIN	0.674 (0.222-2.045) [0.486]		0.996 (0.851-1.167) [0.966]		0.962 (0.778-1.188) [0.717]	0.836 (0.559-1.25) [0.384]	1.166 (0.911-1.493) [0.223]	0.971 (0.878-1.072) [0.557]	0.904 (0.736-1.11) [0.335]	0.951 (0.755-1.198) [0.671]		1.755 (0.725-4.251) [0.213]		0.893 (0.517-1.542) [0.684]	
STREPTOTHRICIN			1.191 (0.900-1.577) [0.222]	1.146 (0.501-2.622) [0.747]	1.124 (0.89-1.42) [0.327]	0.836 (0.448-1.559) [0.573]	1.038 (0.812-1.327) [0.768]	0.832 (0.616-1.126) [0.234]	0.949 (0.784-1.149) [0.595]			0.503 (0.167-1.512) [0.221]			
SULFONAMIDE			0.894 (0.718-1.114) [0.320]	0.878 (0.606-1.271) [0.49]	1.133 (0.853-1.504) [0.388]		1.103 (0.832-1.463) [0.494]	0.964 (0.861-1.079) [0.523]	1.022 (0.888-1.177) [0.756]	1.087 (0.931-1.268) [0.29]		0.867 (0.58-1.295) [0.486]		1.015 (0.474-2.171) [0.97]	
TETRACYCLINE		1.052 (0.491-2.254) [0.896]	0.997 (0.820-1.213) [0.978]	0.716 (0.358-1.431) [0.345]	1.084 (0.904-1.301) [0.383]	1.231 (0.842-1.798) [0.284]	1.036 (0.837-1.283) [0.744]	1.067 (0.968-1.176) [0.189]	0.842 (0.674-1.052) [0.131]	0.952 (0.742-1.221) [0.697]	0.946 (0.587-1.523) [0.818]	0.929 (0.595-1.45) [0.745]		1.384 (0.518-3.699) [0.517]	
BACITRACIN			0.7322 (0.295-1.812) [0.500]						1.035 (0.749-1.43) [0.836]						

ARG [a,b]	Amikacin	Amoxicillin	Cefepime	Cefpodoxime	CIPROfloxacin	DAPTOmycin	Ertapenem	Levofloxacin	Linezolid	Meropenem	Metronidazole	Piperacillin/ Tazobactam	Trimethoprim/ Sulfamethoxazole	Tigecycline	Vancomycin
LINCOSAMIDE/ OXAZOLIDINONES/ PHENICOL/ PLEUROMUTILIN/STREPTOGRAMIN	0.718 (0.260-1.982) [0.523]		0.975 (0.817-1.163) [0.779]	0.917 (0.618-1.363) [0.669]	0.878 (0.706-1.093) [0.245]	0.25 (0.039-1.615) [0.145]	1.057 (0.867-1.289) [0.583]	0.887 (0.762-1.033) [0.124]	1.112 (0.954-1.296) [0.174]	0.967 (0.833-1.123) [0.663]	1.023 (0.762-1.373) [0.881]	0.925 (0.541-1.581) [0.775]		1.098 (0.67-1.799) [0.711]	
BETA-LACTAM	1.655 (0.594-4.611) [0.335]		0.982 (0.826-1.169) [0.842]	1.205 (0.849-1.711) [0.296]	0.853 (0.634-1.146) [0.291]	1.083 (0.748-1.567) [0.674]	0.99 (0.8-1.225) [0.924]	1.061 (0.963-1.168) [0.231]	0.922 (0.732-1.161) [0.488]	1.298 (0.711-2.37) [0.395]	0.952 (0.591-1.531) [0.838]			1.179 (0.605-2.299) [0.629]	
BLEOMYCIN						0.543 (0.105-2.795) [0.465]		1.017 (0.799-1.294) [0.894]	0.587 (0.187-1.847) [0.363]						
COLISTIN		1.574 (0.611-4.054) [0.347]			1.026 (0.774-1.359) [0.86]			1.067 (0.902-1.261) [0.449]	0.625 (0.203-1.922) [0.412]			1.143 (0.733-1.783) [0.556]			
FOSFOMYCIN	1.525 (0.420-5.542) [0.522]	1.857 (0.432-7.976) [0.405]	0.784 (0.438-1.405) [0.414]	0.933 (0.654-1.331) [0.702]	1.389 (0.956-2.016) [0.084]		0.977 (0.787-1.213) [0.832]	1.079 (0.958-1.217) [0.21]	0.966 (0.768-1.217) [0.771]	1.251 (0.864-1.811) [0.235]		1.043 (0.71-1.53) [0.832]			
FOSMIDOMYCIN								1.02 (0.855-1.217) [0.827]	0.797 (0.488-1.301) [0.365]	0.925 (0.627-1.364) [0.695]		0.851 (0.468-1.548) [0.597]		1.288 (0.616-2.694) [0.501]	

[a] Odds ratios (95% Confidence Interval) [p-value]

[b] NA indicates too small sample size
